# Methyl 2-(2-hydroxy­acetamido)benzoate

**DOI:** 10.1107/S1600536810009451

**Published:** 2010-03-24

**Authors:** Samina Alam, Sadaf Saeed, Andreas Fischer, Naeema Khan

**Affiliations:** a Department of Chemistry, Quaid-i-Azam University, Islamabad, Pakistan; b Inorganic Chemistry, School of Chemical Science and Engineering, Royal Institute of Technology (KTH), 100 44 Stockholm, Sweden

## Abstract

The title compound, C_10_H_11_NO_4_, was formed from 4,1-benzoxazepine-2,5(1*H*,3*H*)-dione and ammonia gas. Intra­molecular hydrogen bonding is present between the amide N—H group and the carbonyl O atom of the ester group. The crystal structure features inter­molecular O—H⋯O hydrogen bonds.

## Related literature

For the pharmagological activity of different quinazolinones, see: Kenichi *et al.* (1985 ▶); Lyle (1985*a* ▶,*b* ▶); Mhaske & Argade (2006 ▶); Xia *et al.* (2001 ▶). For details of the synthesis, see: Iacobelli *et al.* (1965 ▶); Uskokovic *et al.* (1964 ▶).
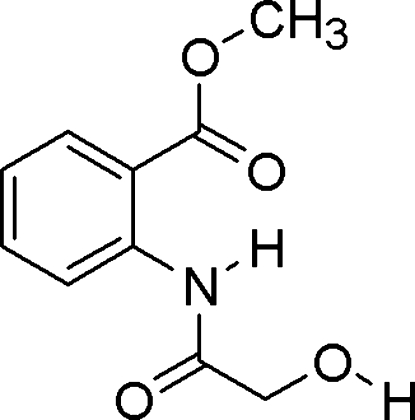


## Experimental

### 

#### Crystal data

C_10_H_11_NO_4_
*M* *_r_* = 209.20
Orthorhombic, 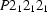

*a* = 3.938 (2) Å
*b* = 8.808 (4) Å
*c* = 27.94 (4) Å
*V* = 969.1 (15) Å^3^
*Z* = 4
Mo *K*α radiation
μ = 0.11 mm^−1^
*T* = 299 K
0.60 × 0.13 × 0.07 mm

#### Data collection

Nonius KappaCCD diffractometer
7794 measured reflections
1107 independent reflections
846 reflections with *I* > 2σ(*I*)
*R*_int_ = 0.104

#### Refinement

*R*[*F* ^2^ > 2σ(*F* ^2^)] = 0.051
*wR*(*F* ^2^) = 0.127
*S* = 1.12
1107 reflections
137 parameters
H-atom parameters constrained
Δρ_max_ = 0.18 e Å^−3^
Δρ_min_ = −0.21 e Å^−3^

### 

Data collection: *COLLECT* (Nonius, 1999 ▶); cell refinement: *DIRAX* (Duisenberg, 1992 ▶); data reduction: *EVALCCD* (Duisenberg *et al.*, 2003 ▶); program(s) used to solve structure: *SHELXS97* (Sheldrick, 2008 ▶); program(s) used to refine structure: *SHELXL97* (Sheldrick, 2008 ▶); molecular graphics: *DIAMOND* (Brandenburg, 2006 ▶); software used to prepare material for publication: *publCIF* (Westrip, 2010 ▶).

## Supplementary Material

Crystal structure: contains datablocks global, I. DOI: 10.1107/S1600536810009451/om2318sup1.cif

Structure factors: contains datablocks I. DOI: 10.1107/S1600536810009451/om2318Isup2.hkl

Additional supplementary materials:  crystallographic information; 3D view; checkCIF report

## Figures and Tables

**Table 1:** Hydrogen-bond geometry (Å, °)

*D*—H⋯*A*	*D*—H	H⋯*A*	*D*⋯*A*	*D*—H⋯*A*
N1—H1*N*⋯O3	0.90	1.95	2.669 (4)	136
O1—H1⋯O2^i^	0.90	1.91	2.758 (4)	157

Symmetry code: (i) 
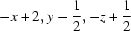
.

**Fig. 1.**
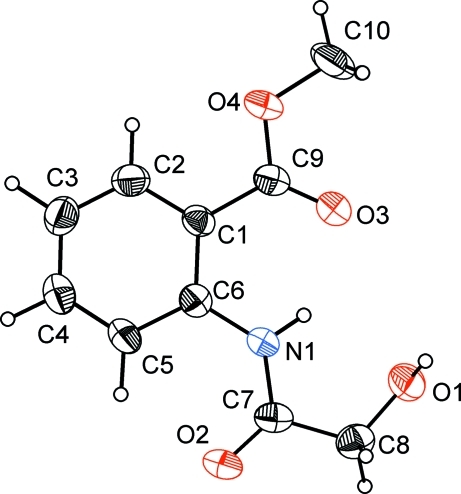
Ellipsoid plot.

**Fig. 2.**
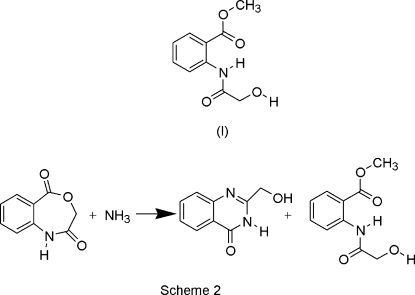
The prepapration of the title compound.

## supplementary crystallographic information

### Comment

4,1-Benzoxazepin-2,5-diones are synthetic heterocyclic compounds that can be converted to quinazolinones which have a broad range of pharmacological activities (Xia *et al.*, 2001; Kenichi *et al.*, 1985; Lyle, 1985*a*,*b*; Mhaske & Argade, 2006). The title compound (I) (Fig. 1) was formed in a ring-cleaving reaction of 4,1-benzoxazepin-2,5-dione and gaseous ammonia (Fig. 2), instead of the expected ring contraction product *i.e.*quinazolinone. The crystal packing of the title compound is stabilized by intermolecular O–H···O bonding. Additionally, intramolecular N–H···O bonds are present.

### Experimental

4,1-benzoxazepin-2,5(1*H*,3*H*)-dione was prepared from the corresponding 2-[(2-chloroethanoyl)amino]benzoic acid (Iacobelli *et al.*, 1965). Ammonia gas was passed through the suspension of 4,1-benzoxazepin-2,5(1*H*,3*H*)-dione (3 g, 0.0169 mole) in dry methanol (400 ml) for three hours and kept at room temperature for seven days. After workup according to reported procedure (Uskokovic *et al.*, 1964) the residue was collected and recrystallized from methanol to give the title compound (I), m.p. 161 °C, yield 31%, *R_f_* 0.76 acetone / benzene (3:7). The undissolved part was recrystallized from hot water to give 2-(1-hydroxymethyl)-4(3*H*)-quinazolinone, m.p. 214 °C; yield 16%, *R_f_* 0.35 acetone / benzene (3:7).

### Refinement

Due to the absence of significant anomalous dispersion, 662 Friedel pairs were merged prior to refinement. H atoms attached to the phenyl group and the methylene group were located in the Fourier map (C–H=0.98–1.04 Å (aromatic), C–H=0.96–1.06 Å (methylene)). All other H atoms were placed at calculated positions (C–H = 0.96 (methyl), N – H=0.90 Å, O–H = 0.90 Å). All atoms were refined as riding on the respective carrier atom.

### Crystal data

**Table:**

C_10_H_11_NO_4_	*F*(000) = 440
*M**_r_* = 209.20	*D*_x_ = 1.434 Mg m^−^^3^
Orthorhombic, *P*2_1_2_1_2_1_	Mo *K*α radiation, λ = 0.71073 Å
Hall symbol: P 2ac 2ab	Cell parameters from 20 reflections
*a* = 3.938 (2) Å	θ = 5.9–19.1°
*b* = 8.808 (4) Å	µ = 0.11 mm^−^^1^
*c* = 27.94 (4) Å	*T* = 299 K
*V* = 969.1 (15) Å^3^	Needle, colourless
*Z* = 4	0.60 × 0.13 × 0.07 mm

### Data collection

**Table:**

Nonius KappaCCD diffractometer	*R*_int_ = 0.104
Radiation source: fine-focus sealed tube	θ_max_ = 25.5°, θ_min_ = 4.6°
φ and ω scans	*h* = −4→4
7794 measured reflections	*k* = −10→10
1107 independent reflections	*l* = −33→33
846 reflections with *I* > 2σ(*I*)	

### Refinement

**Table:**

Refinement on *F*^2^	Primary atom site location: structure-invariant direct methods
Least-squares matrix: full	Secondary atom site location: difference Fourier map
*R*[*F*^2^ > 2σ(*F*^2^)] = 0.051	H-atom parameters constrained
*wR*(*F*^2^) = 0.127	*w* = 1/[σ^2^(*F*_o_^2^) + (0.0491*P*)^2^ + 0.4762*P*] where *P* = (*F*_o_^2^ + 2*F*_c_^2^)/3
*S* = 1.12	(Δ/σ)_max_ < 0.001
1107 reflections	Δρ_max_ = 0.18 e Å^−^^3^
137 parameters	Δρ_min_ = −0.21 e Å^−^^3^
0 restraints	

### Special details

**Table:**

Geometry. All e.s.d.'s (except the e.s.d. in the dihedral angle between two l.s. planes) are estimated using the full covariance matrix. The cell e.s.d.'s are taken into account individually in the estimation of e.s.d.'s in distances, angles and torsion angles; correlations between e.s.d.'s in cell parameters are only used when they are defined by crystal symmetry. An approximate (isotropic) treatment of cell e.s.d.'s is used for estimating e.s.d.'s involving l.s. planes.
Refinement. Refinement of *F*^2^ against ALL reflections. The weighted *R*-factor *wR* and goodness of fit *S* are based on *F*^2^, conventional *R*-factors *R* are based on *F*, with *F* set to zero for negative *F*^2^. The threshold expression of *F*^2^ > σ(*F*^2^) is used only for calculating *R*-factors(gt) *etc*. and is not relevant to the choice of reflections for refinement. *R*-factors based on *F*^2^ are statistically about twice as large as those based on *F*, and *R*- factors based on ALL data will be even larger.

### Fractional atomic coordinates and isotropic or equivalent isotropic displacement parameters (Å^2^)

**Table:**

	*x*	*y*	*z*	*U*_iso_*/*U*_eq_	
C1	0.9247 (10)	0.4009 (4)	0.09627 (12)	0.0331 (9)	
C2	1.0237 (11)	0.3866 (4)	0.04884 (13)	0.0425 (10)	
C3	1.1821 (12)	0.5015 (5)	0.02483 (13)	0.0467 (11)	
C4	1.2470 (12)	0.6356 (5)	0.04868 (14)	0.0468 (11)	
C5	1.1577 (11)	0.6541 (4)	0.09563 (13)	0.0421 (10)	
C6	0.9931 (9)	0.5380 (4)	0.12037 (12)	0.0338 (9)	
C7	0.9656 (11)	0.6678 (4)	0.19938 (13)	0.0382 (10)	
C8	0.8338 (11)	0.6424 (4)	0.24887 (13)	0.0429 (10)	
C9	0.7492 (10)	0.2746 (4)	0.12057 (13)	0.0368 (9)	
C10	0.5821 (14)	0.0186 (4)	0.11595 (17)	0.0627 (14)	
N1	0.8981 (9)	0.5541 (3)	0.16834 (10)	0.0367 (8)	
O1	0.6276 (7)	0.5119 (3)	0.25294 (8)	0.0482 (8)	
O2	1.1235 (9)	0.7833 (3)	0.18947 (9)	0.0545 (9)	
O3	0.6172 (9)	0.2797 (3)	0.15945 (10)	0.0546 (8)	
O4	0.7495 (8)	0.1475 (3)	0.09478 (9)	0.0529 (8)	
H2	0.9631	0.2943	0.0312	0.051*	
H3	1.2568	0.4854	−0.0104	0.056*	
H4	1.3879	0.7148	0.0317	0.056*	
H5	1.2063	0.7559	0.1121	0.051*	
H8A	0.6860	0.7378	0.2595	0.051*	
H8B	1.0239	0.6257	0.2695	0.051*	
H10A	0.7049	−0.0132	0.1439	0.075*	
H10B	0.5749	−0.0631	0.0932	0.075*	
H10C	0.3548	0.0462	0.1248	0.075*	
H1N	0.7402	0.4862	0.1774	0.044*	
H1	0.7463	0.4332	0.2647	0.058*	

### Atomic displacement parameters (Å^2^)

**Table:**

	*U*^11^	*U*^22^	*U*^33^	*U*^12^	*U*^13^	*U*^23^
C1	0.038 (2)	0.0260 (18)	0.0351 (17)	0.0004 (18)	−0.0033 (18)	0.0036 (15)
C2	0.050 (3)	0.037 (2)	0.041 (2)	−0.002 (2)	−0.003 (2)	−0.0059 (17)
C3	0.055 (3)	0.049 (2)	0.0360 (18)	0.002 (2)	0.004 (2)	0.002 (2)
C4	0.052 (3)	0.043 (2)	0.045 (2)	−0.005 (2)	0.005 (2)	0.0112 (19)
C5	0.050 (3)	0.032 (2)	0.044 (2)	−0.008 (2)	0.002 (2)	0.0064 (18)
C6	0.033 (2)	0.034 (2)	0.0344 (17)	−0.0008 (18)	−0.0028 (17)	0.0025 (16)
C7	0.042 (2)	0.0298 (19)	0.043 (2)	0.002 (2)	−0.0050 (18)	−0.0055 (17)
C8	0.046 (2)	0.040 (2)	0.0422 (19)	0.005 (2)	−0.001 (2)	−0.0041 (18)
C9	0.039 (2)	0.0304 (19)	0.0405 (19)	−0.0024 (19)	−0.005 (2)	−0.0016 (17)
C10	0.075 (4)	0.030 (2)	0.083 (3)	−0.015 (3)	0.006 (3)	0.001 (2)
N1	0.0444 (19)	0.0288 (15)	0.0370 (15)	−0.0097 (17)	0.0031 (16)	−0.0025 (13)
O1	0.060 (2)	0.0346 (14)	0.0502 (14)	0.0024 (16)	0.0045 (16)	0.0060 (13)
O2	0.079 (2)	0.0317 (14)	0.0528 (15)	−0.0158 (18)	0.0039 (17)	−0.0075 (13)
O3	0.079 (2)	0.0363 (14)	0.0487 (15)	−0.0143 (18)	0.0146 (17)	−0.0024 (13)
O4	0.073 (2)	0.0281 (14)	0.0577 (16)	−0.0129 (15)	0.0110 (17)	−0.0084 (13)

### Geometric parameters (Å, °)

**Table:**

C1—C2	1.387 (5)	C9—O4	1.331 (4)
C1—C6	1.409 (5)	C10—O4	1.440 (5)
C1—C9	1.475 (5)	C2—H2	0.9802
C2—C3	1.365 (5)	C3—H3	1.0375
C3—C4	1.380 (6)	C4—H4	1.0097
C4—C5	1.368 (5)	C5—H5	1.0250
C5—C6	1.394 (5)	C8—H8A	1.0646
C6—N1	1.399 (5)	C8—H8B	0.9558
C7—O2	1.224 (4)	C10—H10A	0.9600
C7—N1	1.351 (5)	C10—H10B	0.9600
C7—C8	1.493 (5)	C10—H10C	0.9600
C8—O1	1.412 (5)	N1—H1N	0.8989
C9—O3	1.205 (4)	O1—H1	0.8986
			
C2—C1—C6	118.7 (3)	C2—C3—H3	119.7
C2—C1—C9	120.2 (3)	C4—C3—H3	121.5
C6—C1—C9	121.1 (3)	C5—C4—H4	120.6
C3—C2—C1	122.1 (4)	C3—C4—H4	117.8
C2—C3—C4	118.8 (3)	C4—C5—H5	119.0
C5—C4—C3	121.1 (4)	C6—C5—H5	120.4
C4—C5—C6	120.5 (4)	O1—C8—H8A	107.8
C5—C6—N1	121.7 (3)	C7—C8—H8A	109.4
C5—C6—C1	118.8 (3)	O1—C8—H8B	106.0
N1—C6—C1	119.5 (3)	C7—C8—H8B	108.0
O2—C7—N1	124.8 (3)	H8A—C8—H8B	112.4
O2—C7—C8	120.7 (3)	O4—C10—H10A	109.5
N1—C7—C8	114.5 (3)	O4—C10—H10B	109.5
O1—C8—C7	113.3 (3)	H10A—C10—H10B	109.5
O3—C9—O4	121.4 (3)	O4—C10—H10C	109.5
O3—C9—C1	126.0 (3)	H10A—C10—H10C	109.5
O4—C9—C1	112.6 (3)	H10B—C10—H10C	109.5
C7—N1—C6	129.6 (3)	C7—N1—H1N	116.6
C9—O4—C10	116.1 (3)	C6—N1—H1N	112.8
C3—C2—H2	118.6	C8—O1—H1	111.0
C1—C2—H2	119.1		

### Hydrogen-bond geometry (Å, °)

**Table:**

*D*—H···*A*	*D*—H	H···*A*	*D*···*A*	*D*—H···*A*
N1—H1N···O3	0.90	1.95	2.669 (4)	136
O1—H1···O2^i^	0.90	1.91	2.758 (4)	157

Symmetry codes: (i) −*x*+2, *y*−1/2, −*z*+1/2.
